# Preseason isokinetic strength imbalance and hamstring injury in male professional rugby players: a prospective cohort study

**DOI:** 10.3389/fspor.2026.1961372

**Published:** 2026-10-28

**Authors:** Maxime Grolier, Mathilde Pelletier Visa, Marc Julia, Dominique Morand, Bruno Pereira, Céline Lambert, Emmanuel Coudeyre

**Affiliations:** 1 Service de Médecine Physique et de Réadaptation, CHU Clermont-Ferrand, Université Clermont Auvergne, INRAE, UNH, Clermont-Ferrand, France; 2 Service de Médecine Physique et de Réadaptation, CHRU Montpellier, Montpellier, France; 3 Secteur Biométrie et Médico-économie, Direction de la Recherche Clinique et de l’Innovation, CHU Clermont-Ferrand, Clermont-Ferrand, France

**Keywords:** epidemiology, injury prevention, isokinetic, muscle injury, rugby

## Abstract

**Background:**

Hamstring injuries are among the most common muscle injuries in team ball sports. Hamstring muscle strength deficits have been identified as potential intrinsic risk factors for hamstring injury.

**Objectives:**

To determine the cumulative incidence of hamstring injuries in male professional “Top 14” rugby players during one season and to investigate the associations between preseason isokinetic strength imbalance and subsequent hamstring injury.

**Methods:**

This prospective cohort study included 134 male professional rugby players from four French “Top 14” clubs recruited during the 2010–2011 and 2011–2012 seasons. The four participating clubs were selected from the 14 eligible “Top 14” clubs, and player participation was voluntary. Preseason isokinetic and muscle extensibility assessments were performed, and playing position and previous hamstring injury history were recorded. A preseason isokinetic strength imbalance was defined as the presence of at least one prespecified abnormality in bilateral hamstring symmetry, conventional ratio, or mixed/functional ratio. Players were followed prospectively for 12 months. Training and match exposure time were not collected. Associations with subsequent hamstring injury were assessed using multivariable logistic regression with adjustment for clustering by club.

**Results:**

During the 12-month follow-up, 40 of 134 players sustained at least one hamstring injury, corresponding to a cumulative incidence of 29.9% (95% CI: 22.2–38.4). The cumulative incidence was 21.0% (13/62; 95% CI: 11.6–33.2) in players without a preseason isokinetic imbalance and 37.5% (27/72; 95% CI: 26.3–49.7) in those with an imbalance. In the single-predictor mixed-effects model accounting for club-level clustering, players who met at least one predefined criterion for preseason isokinetic imbalance had higher odds of hamstring injury than players who met none of these criteria, although the association did not reach statistical significance (OR 2.12, 95% CI 0.93–4.83, *p* = 0.073). After adjustment for previous hamstring injury and playing position, the estimated OR increased to 2.56 (95% CI: 1.07–6.12, *p* = 0.034).

**Conclusion:**

In this cohort of male professional rugby players, preseason isokinetic strength imbalance was associated with higher odds of subsequent hamstring injury after adjustment for previous hamstring injury and playing position. However, the data were collected in 2011–2012, and the composite classification showed limited individual level discrimination. These findings should therefore be considered exploratory and require replication in contemporary professional rugby union cohorts using standardized injury definitions, exposure-adjusted analyses, and validated rugby-specific classification thresholds before their clinical relevance can be established.

## Introduction

Hamstring injuries are among the most common muscle injuries in team ball sports, such as football (1–3), Australian football (4, 5) and American football (6) and rugby (7–9). In rugby union, a recent systematic review reported that hamstring injuries may account for up to 15% of all injuries (10). In professional rugby union, Brooks et al. reported hamstring injury incidences of 0.27 per 1,000 training hours and 5.6 per 1,000 match hours, with recurrent injuries accounting for 23% of hamstring injuries and resulting in greater time loss than first-time injuries (8). Hamstring injury is multifactorial and involves both extrinsic factors related to sport participation and intrinsic factors related to individual athlete characteristics (10, 11). Other intrinsic factors that have been investigated include reduced muscle flexibility (12) and hamstring muscle strength deficits (13).

Isokinetic dynamometry is a reliable laboratory method for quantifying dynamic thigh muscle strength and enables standardized assessment of unilateral and bilateral strength characteristics (14, 15). Its use and reproducibility for knee-joint assessment have been widely investigated. However, the clinical validity of isokinetic measures for predicting subsequent hamstring injury remains uncertain, with inconsistent findings across sporting populations (16–18).

In a prospective study of 462 professional football players, Croisier et al. (19) reported a significantly higher risk of hamstring injuries among players with untreated preseason muscle strength imbalances compared with those without imbalances [relative risk 4.66, 95% confidence interval (CI): 2.01–10.80]. Among players whose isokinetic parameters were subsequently normalized, the risk was no longer significantly different from that observed in players without preseason imbalances (relative risk: 1.43, 95% CI: 0.44–4.71). Their classification was based on predefined thresholds for bilateral hamstring asymmetry and conventional and mixed/functional hamstring-to-quadriceps ratios. However, these thresholds were derived from professional football players and have not been externally validated in professional rugby union players (20).

More broadly, the transferability of football-derived isokinetic thresholds to professional rugby union warrants consideration. The application of this technique and its reproducibility for the knee joint have been widely validated in the literature (15, 21, 22). Isokinetic dynamometry provides a standardized method for assessing dynamic muscle strength under controlled angular velocities, although its applications and limitations should be carefully considered (23). At the time of data collection (2011–2012), rugby-specific isokinetic reference values were not available to define these abnormalities. The criteria described by Croisier et al. were therefore used as the predefined classification framework in the present study (22). Rugby-specific isokinetic reference data have subsequently been published in French professional rugby union players, suggesting that sport-specific reference values may result in a different classification of strength abnormalities in this population (24). The potential impact of applying such rugby-specific thresholds should therefore be considered when interpreting the present findings.

Evidence regarding the ability of preseason isokinetic strength measures to predict subsequent hamstring injury remains inconsistent. Bennell et al. reported that preseason isokinetic strength testing did not predict subsequent hamstring injury in Australian Rules football players (16). These findings contrast with those reported by Croisier et al. and illustrate the uncertainty regarding the prognostic value of preseason isokinetic assessment across sporting populations (19).

Given the frequency and recurrence of hamstring injuries, assessment of hamstring extensibility and muscle strength may be relevant for identifying potentially modifiable characteristics. However, the identification of an isokinetic abnormality does not in itself establish that correcting the abnormality will reduce subsequent injury occurrence. Prospective studies have investigated preseason hamstring strength and subsequent injury occurrence in rugby union, including eccentric knee-flexor strength assessed during the Nordic hamstring exercise (18). However, to our knowledge, no published prospective study has specifically evaluated the association between preseason isokinetic muscle strength and thigh muscle extensibility measures and subsequent hamstring injury in professional rugby union players (10). Importantly, evidence from other sports indicates that the predictive value of isolated strength measures for subsequent hamstring injury occurrence at the individual level is limited. A systematic review and meta-analysis of 12 prospective studies including 2,912 athletes found limited predictive validity of isokinetic strength assessment, with most strength measures showing no consistent association with subsequent hamstring injury (17).

Similarly, a prospective cohort study of 413 professional football players found that a comprehensive battery of isokinetic and eccentric strength measures had limited clinical value for predicting future hamstring injury (25). Conversely, prospective evidence in rugby union has reported an association between-limb eccentric knee-flexor strength imbalance and subsequent hamstring injury, illustrating the heterogeneity of the available evidence (18). Furthermore, systematic reviews indicate that hamstring injury is multifactorial and that individual factors should not be interpreted in isolation (11). Taken together, these findings indicate that associations between preseason strength characteristics and subsequent hamstring injury may vary according to the sport, the strength measure assessed, and the population studied. Moreover, demonstrating an association between a screening measure and injury occurrence does not establish its value as an individual injury-prediction tool (26). Therefore, the objective of the present study was not to validate isokinetic testing as a standalone individual prediction tool, but to investigate whether preseason isokinetic muscle strength and thigh muscle extensibility measures are associated with subsequent hamstring injury in professional rugby union players.

## Methods

### Study design

This prospective cohort study was conducted with a 12-month follow-up period to determine the cumulative incidence of hamstring injuries in male professional rugby players. Baseline isokinetic assessments were conducted either during the final two weeks of the 2010–2011 season or during the first three weeks of the 2011–2012 season, depending on the preseason schedule of each participating club. The individual baseline was defined as the date of the isokinetic assessment. This manuscript was prepared in accordance with the STrengthening the Reporting of OBservational studies in Epidemiology (STROBE) guidelines for reporting observational studies (27).

### Primary objective

The primary objective was to determine the cumulative incidence of hamstring injuries in male professional rugby players over the 12-month follow-up period.

### Secondary objectives

Secondary objectives were to investigate the associations between preseason isokinetic muscle strength characteristics, thigh muscle extensibility, previous hamstring injury, playing position, and the subsequent occurrence of hamstring injury.

### Injury ascertainment

During follow-up, injuries involving the thigh and knee regions were prospectively recorded by the club medical staff of each participating club using a study-specific injury questionnaire. The questionnaire was developed specifically for this study and was not formally validated, and no formal assessment of its reliability was performed (Supplementary File S1).

Hamstring injuries were identified based on clinical examination by the club medical staff of the participating clubs. When clinically indicated, imaging examinations, including ultrasound and/or magnetic resonance imaging, were performed to support the diagnosis. Imaging was therefore not systematically performed for all hamstring injuries. Club medical staff had access to players' preseason isokinetic results and group allocation during follow-up; therefore, injury ascertainment was not blinded to baseline isokinetic status.

Similarly, the relationships between muscle injury and muscle strength parameters (assessed with an isokinetic device) during the preseason, on the one hand, and the presence of a previous injury, on the other hand, were studied. The associations of these elements with the occurrence of hamstring injury were evaluated as has been done in other samples (19).

### Study procedures

At the inclusion visit, players from each participating professional club received detailed information about the study and provided written non-objection form. The following data were then collected: field position, weight, height, and medical and surgical history. Muscle extensibility was assessed before the warm-up by the physician of each participating club.

For the hamstrings, the Straight Leg Raise test was performed with the player in the supine position, with the knee maintained in full extension and the ankle relaxed. The tested lower limb was passively and progressively raised into hip flexion until the onset of pain or a mechanical limitation. The angle of the lower limb was measured in degrees using an inclinometer. The test was repeated on the contralateral side. Two measurements were obtained for each side, and the mean of the two measurements obtained on each side was used for the analysis.

For the quadriceps, muscle extensibility was assessed with the player in the prone position. The examiner passively flexed the knee, bringing the heel toward the buttock. The heel-to-buttock distance was measured in centimeters using a measuring tape, with a greater distance indicating lower quadriceps extensibility. The assessment was performed bilaterally. Two measurements were obtained for each side, and the mean value of the two measurements obtained on each side was retained for analysis. They then warmed up on a cycle ergometer for 10 min and performed a maximal isokinetic test for both thighs. Isokinetic testing is used in professional sports settings for the assessment of lower-limb muscle strength, including in rugby.

During the 12-month prospective follow-up, injury surveillance was performed by the club medical staff as part of routine clinical practice. Club medical staff were responsible for identifying and documenting new thigh muscle injuries occurring after baseline.

When an isokinetic imbalance was identified, the results were communicated to the clubs. Corrective strength training was implemented for at least some players during the follow-up period as part of their subsequent clinical and performance management. The nature, timing and adherence to these corrective interventions were not systematically recorded. For each suspected injury, information was collected on the injured structure and side, injury type, circumstances and phase of play, field conditions, and the player's position at the time of injury.

Data were anonymized throughout the protocol. Investigators involved in data processing had access only to anonymized data.

### Participants

The medical staff of the 14 “Top 14” clubs competing during the 2011–2012 season were invited to participate in the study. Based on archived team rosters and original study documentation, these 14 clubs comprised approximately 490 professional players. This figure represents the number of players identified on the club rosters and not the number of players individually screened or confirmed as eligible. Two clubs did not have access to an isokinetic dynamometer and four clubs declined participation. The remaining eight clubs, comprising approximately 280 players, agreed to participate.

No individual player refusal was documented among these eight participating clubs. However, individual-level screening records were not available for all rostered players; therefore, the exact number of players who fulfilled all eligibility criteria cannot be determined retrospectively.

Four of the eight participating clubs were subsequently not retained in the final analysis because consistent data collection could not be ensured due to incomplete isokinetic data and/or changes in medical staff or player composition during the study period. Consequently, four clubs comprising 134 professional rugby players were retained in the final cohort.

Participation was voluntary and no financial compensation was provided.

Eligibility criteria were: age ≥18 years; professional rugby union player status; ability to safely perform hamstring and quadriceps muscle testing; receipt of study information; and provision of written non-objection form.

Players with a recent quadriceps or hamstring muscle injury were excluded from isokinetic testing. An injury was considered recent if the player had not yet returned to opposition training or match play for at least two weeks at the time of the preseason assessment. This criterion was applied to avoid maximal isokinetic testing in players who had not yet achieved a sufficient return to sport following a recent muscle injury.

Previous hamstring injury was recorded as a history of hamstring injury if the player had sustained a hamstring injury at any time before the preseason assessment and had subsequently returned to opposition training or match play for at least two weeks. No upper time limit was applied to previous hamstring injury; therefore, all documented previous hamstring injuries meeting this recovery criterion were considered in the analysis, irrespective of the time elapsed since the injury.

The investigator reserved the right to stop testing if the player's health status did not allow completion of the protocol. In the event of premature withdrawal from the study, the date and reason were recorded on the player's follow-up form.

### Variables

The primary outcome was the cumulative incidence of hamstring injury over the 12-month follow-up, defined as the proportion of players who sustained at least one hamstring injury during follow-up. The outcome was coded as a binary variable (yes/no) according to whether at least one hamstring injury was recorded during follow-up. Therefore, the analysis focused on the occurrence of at least one recorded hamstring injury per player.

A hamstring injury was defined as a clinically diagnosed injury resulting in more than two weeks of time loss from training and/or competition. The injury questionnaire included items on injury date, recurrence status, contact or non-contact mechanism, phase of play, playing-surface conditions, and diagnostic investigations. However, the corresponding information was not sufficiently complete in the retained analytical dataset to allow reliable episode-level analyses. Standardized injury severity grading was not available.

Secondary variables included baseline parameters such as muscle strength parameters (assessed by isokinetic dynamometry), muscle extensibility parameters, history of hamstring injury, body mass index and playing position. Playing position was categorized into two groups according to standard rugby positional classifications: forwards (positions 1–8) and backs (positions 9–15). Age was not collected because of confidentiality requirements related to the participating professional clubs.

### Assessment of muscle strength

Assessments were performed using a motorized computerized dynamometer (Con-Trex® or Cybex Norm®). Previous studies have directly compared the Con-Trex® and Cybex Norm® dynamometers and reported differences in measured torque values between devices, indicating that absolute measurements obtained with these systems should not be considered fully interchangeable (28, 29). Before each assessment, the dynamometer underwent its standard automatic calibration procedure according to the manufacturer's instructions. Measurements were preceded by a warm-up consisting of pedaling on an ergometric bicycle at 1 W/kg. Participants were seated on the dynamometer with the hip flexed at 85°, and their body was stabilized using straps around the thigh, waist, and chest to minimize compensatory movements. The gravitational effect of the dynamometer's lever arm and the lower leg-segment ensemble was compensated for during the measurements. The isokinetic testing protocol consisted of concentric contractions of the quadriceps and hamstring muscle groups at angular velocities of 60°/s and 240°/s, followed by eccentric testing of the hamstrings at 30°/s and 120°/s. At 60°/s, three familiarization repetitions were followed by three maximal repetitions, while at 240°/s, three familiarization repetitions were followed by five maximal repetitions. Eccentric hamstring testing at 30°/s consisted of three familiarization repetitions, or additional repetitions when necessary, followed by three maximal repetitions; testing was subsequently performed at 120°/s with three familiarization repetitions followed by four maximal repetitions. Eccentric quadriceps testing was not performed. All testing sets were separated by a 1 min rest interval.

Before the maximal testing protocol, participants underwent a standardized isokinetic familiarization procedure at 120°/s, consisting of 6–7 repetitions at submaximal effort followed by 3–4 progressively maximal repetitions. These familiarization repetitions were not recorded for analysis. The participant was seated with the seat raised and the backrest inclined at 85°, and the range of motion was 100° of knee flexion from the maximal active extension position, without recurvatum. The contralateral lower limb remained free during testing. No visual feedback was provided during the test, while verbal encouragement was given by the evaluator. All physical therapists responsible for isokinetic testing received standardized instructions to ensure consistent application of the testing procedure (Supplementary Table S1).

Maximum peak torque was retained for analysis and expressed relative to body mass. Bilateral differences between the right and left limbs were calculated for each muscle group and testing condition. For the present classification, eccentric hamstring asymmetry was assessed at 30°/s. The conventional hamstring/quadriceps (H/Q) ratio was calculated from peak torque values obtained during concentric contractions at the corresponding angular velocities. Because eccentric testing was performed for the hamstrings only, an eccentric conventional H/Q ratio was not calculated. Oral encouragement was given.

### Classification of isokinetic imbalance

In the absence of rugby union-specific consensus thresholds at the time of the study, players were classified into two groups. Group A comprised players who did not meet any of the predefined criteria for isokinetic imbalance, whereas Group B comprised players who met at least one criterion.

- Bilateral hamstring asymmetry: a side-to-side hamstring strength deficit >15% during concentric testing at 60°/s or 240°/s, or during eccentric hamstring testing at 30°/s (19).
- Conventional H/Q ratio: the concentric hamstring peak torque divided by the concentric quadriceps peak torque at the same angular velocity. A ratio <0.45 at 60°/s or <0.55 at 240°/s was considered abnormal (22).
- Mixed (functional) H/Q ratio: the eccentric hamstring peak torque at 30°/s divided by the concentric quadriceps peak torque at 240°/s (Hecc30/Qcon240). A ratio <0.85 was considered abnormal.

Eccentric quadriceps testing was not performed; therefore, an eccentric conventional H/Q ratio could not be calculated.

### Statistical analysis

The sample size was determined according to the expected number of professional rugby players available from the four participating clubs and was intended to provide an estimate of the precision of the primary descriptive endpoint. Assuming a hamstring injury proportion of approximately 15%, a sample of 120–150 players was expected to provide a 95% CI with a half-width of around 6 percentage points (approximately 5.7–6.4 percentage points). No formal sample-size or power calculation was performed for the secondary comparative analyses, which were considered exploratory.

Statistical analysis was performed using Stata software (version 15; StataCorp, College Station, Texas, USA). All tests were two-sided, with an alpha level set at 5%. Categorical data are expressed as the number of players and percentages, and continuous data are expressed as the mean ± standard deviation or median [25^th^; 75^th^ percentiles], according to their statistical distribution. There were no missing data for any of the variables included in the analyses; therefore, no imputation or other methods for handling missing data were required. Comparisons between groups A and B were carried out via the following statistical tests: the chi-square test or Fisher's exact test for categorical variables and Student's t test or the Mann‒Whitney *U* test for quantitative variables. The cumulative incidence of hamstring injuries was reported with a 95% CI estimated by a binomial distribution. The factors associated with hamstring injuries were studied via generalized linear mixed models (with a logit link function) to consider a cluster (club) effect. A random intercept for club was included to account for within-club clustering. Models were estimated by maximum likelihood using adaptive Gauss-Hermite quadrature with 12 integration points. No convergence difficulties were encountered. The between-club variance and corresponding intraclass correlation coefficient were estimated. The results are expressed as odds ratios (ORs) and 95% CIs. Finally, a multivariable analysis was performed using the same statistical model. The primary exposure (preseason isokinetic imbalance) was included as a fixed effect, together with previous hamstring injury and playing position, which were selected *a priori* as clinically relevant potential confounders. Given the limited number of injury events, the number of covariates was restricted to limit model complexity and reduce the risk of overfitting (30).

The study was conducted according to a prospectively established protocol. No separate statistical analysis plan was prospectively registered. Some additional analyses and model revisions presented in the current manuscript were conducted during peer-review revision. A subgroup analysis was performed according to previous hamstring injury status. As a sensitivity analysis, the association between preseason isokinetic imbalance and subsequent hamstring injury was also examined using a logistic regression model without a club-level random effect. No adjustment for multiplicity was applied to secondary and exploratory analyses.

### Equity, diversity and inclusion statement

The study population consisted exclusively of male professional rugby union players, reflecting the composition of the professional “Top 14” competition at the time of the study. The research team is diverse in terms of gender. Race, ethnicity, and cultural background were not assessed. No discrimination was made during recruitment, and participation was voluntary among eligible players at participating clubs.

## Results

### Participants

Fourteen eligible “Top 14” clubs were invited to participate, and four clubs participated in the study. A total of 134 players were included in the cohort and followed prospectively for 12 months. No loss to follow-up was reported (Figure 1).

**Figure 1:**
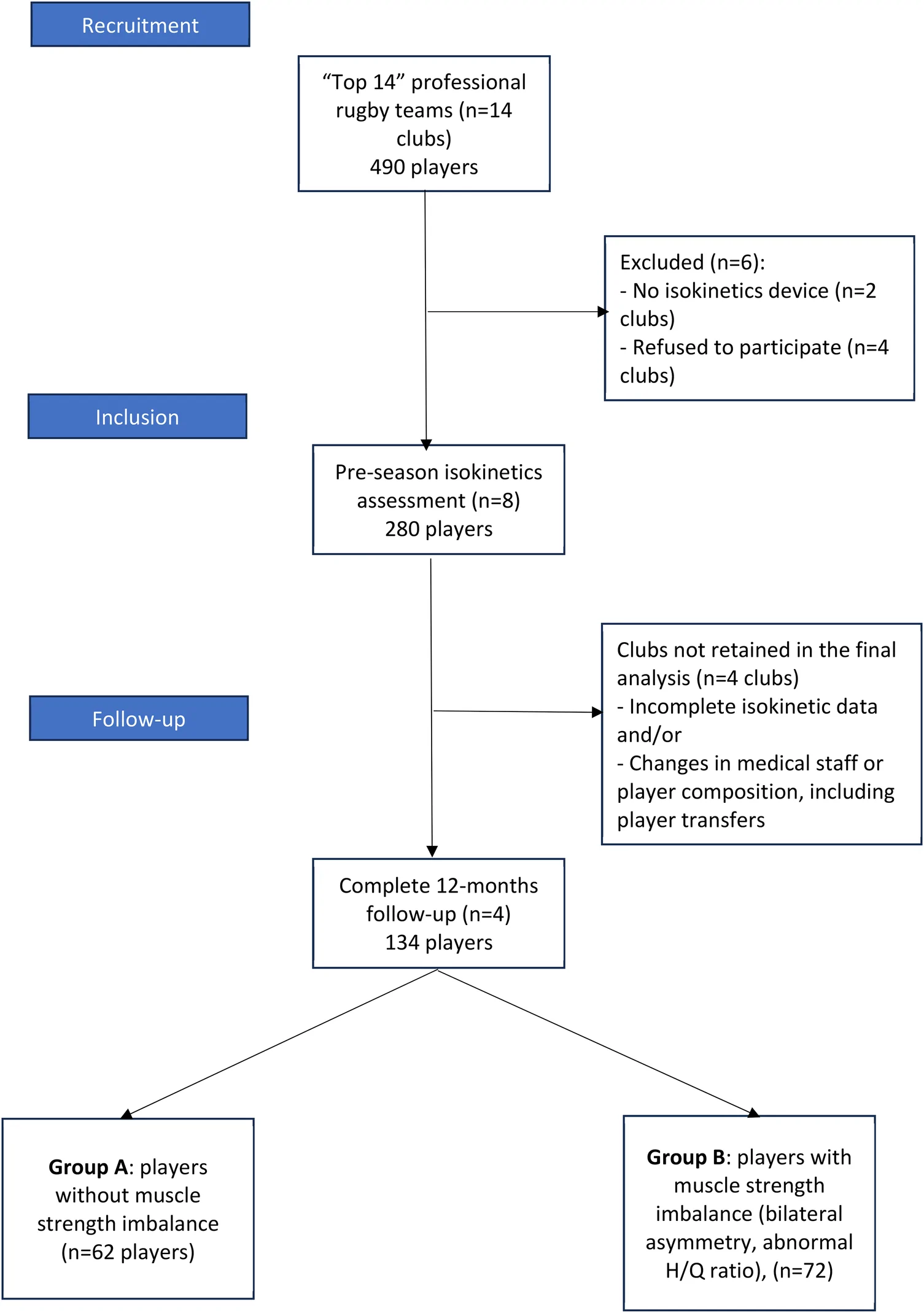
Flowchart of the study. H, hamstring; Q, quadriceps*.*

Group A, comprising players without isokinetic muscle strength imbalance, included 62 players, whereas Group B, comprising players with at least one isokinetic imbalance included 72 players. In this sample, 23.9% of players had a history of previous hamstring injury: 30.6% in Group A and 18.1% in Group B. Characteristics of the participants are presented in Table 1. Among the 134 players, 25 (18.7%) had an abnormal conventional H/Q ratio, 36 (26.9%) had an abnormal mixed H/Q ratio, and 11 (8.2%) showed a bilateral asymmetry greater than 15%. Overall, 72 players (53.7%) had at least one isokinetic abnormality.

**Table 1:** Characteristics of the participants at baseline (*n* = 134).

Characteristics	Total (*n* = 134)	Group A (*n* = 62)	Group B (*n* = 72)
Weight (kg)	102.1 ± 14.3	102.8 ± 13.6	101.5 ± 15.0
Height (cm)	185.7 ± 8.4	186.2 ± 7.7	185.2 ± 9.0
Body mass index (kg/m^2^)	29.6 ± 3.5	29.6 ± 3.0	29.6 ± 3.8
Playing position			
Forwards (1–8)	80 (59.7)	39 (62.9)	41 (56.9)
Backs (9–15)	54 (40.3)	23 (37.1)	31 (43.1)
History of hamstring injury	32 (23.9)	19 (30.6)	13 (18.1)
Extensibility of thigh muscles			
Right hamstring (°)	82.9 ± 10.3	82.3 ± 11.0	83.4 ± 9.6
Left hamstring (°)	81.6 ± 10.3	81.4 ± 11.3	81.7 ± 9.4
Right quadriceps (cm)	8 [0; 12]	6 [2; 12]	9 [0; 13]
Left quadriceps (cm)	8 [0; 13]	6 [2; 13]	9 [0; 13]

Data are presented as number of players (percentages in columns), mean ± standard deviation or median [25th; 75th percentiles]. Group A, players without muscle strength imbalance; Group B, players with muscle strength imbalance.

Peak torque was recorded in absolute values (Nm) and normalized to body mass (Nm/kg). Normalized values are presented in Table 2, while absolute peak torque values are provided in Supplementary Table S2. Overall, Group B showed lower normalized concentric hamstring peak torque at 60°/s and lower eccentric hamstring peak torque at 30°/s than Group A. The left mixed H/Q ratio was also lower in Group B, whereas the other isokinetic parameters showed more limited between-group differences (Table 2).

**Table 2:** Isokinetic characteristics according to preseason imbalance status.

Measures	Total (*n* = 134)	Group A (*n* = 62)	Group B (*n* = 72)	*p*
Peak concentric hamstring torque 60°/s				
Right	1.64 ± 0.41	1.72 ± 0.37	1.58 ± 0.43	0.039
Left	1.65 ± 0.43	1.73 ± 0.39	1.58 ± 0.46	0.043
Peak concentric quadriceps torque 60°/s				
Right	2.66 ± 0.72	2.73 ± 0.65	2.60 ± 0.77	0.275
Left	2.62 ± 0.68	2.67 ± 0.68	2.57 ± 0.69	0.394
Peak concentric hamstring torque 240°/s				
Right	1.28 ± 0.32	1.29 ± 0.32	1.27 ± 0.32	0.617
Left	1.29 ± 0.38	1.31 ± 0.39	1.27 ± 0.37	0.523
Peak concentric quadriceps torque 240°/s				
Right	1.80 ± 0.46	1.79 ± 0.40	1.82 ± 0.50	0.691
Left	1.79 ± 0.44	1.76 ± 0.45	1.82 ± 0.43	0.377
Peak eccentric hamstring torque 30°/s				
Right	2.09 ± 0.50	2.21 ± 0.47	2.00 ± 0.50	0.012
Left	2.08 ± 0.60	2.26 ± 0.51	1.92 ± 0.62	<0.001
Bilateral difference in peak torque, %				
Hamstring at 60°/s	−0.1 ± 13.9	−0.3 ± 7.1	0.0 ± 17.9	0.874
Quadriceps at 60°/s	1.2 ± 15.6	2.5 ± 12.8	0.2 ± 17.7	0.378
Hamstring at 240°/s	0.3 ± 14.8	−0.0 ± 13.6	0.6 ± 15.8	0.806
Quadriceps at 240°/s	0.5 ± 13.1	2.5 ± 11.8	−1.1 ± 14.0	0.109
Hamstring in eccentric at 30°/s	1.6 ± 14.9	−1.9 ± 7.1	4.7 ± 18.8	0.007
Conventional H/Q ratio 60°/s				
Right	0.63 ± 0.15	0.64 ± 0.10	0.63 ± 0.19	0.710
Left	0.64 ± 0.14	0.67 ± 0.15	0.62 ± 0.12	0.070
Mixed H/Q ratio				
Right	1.22 ± 0.43	1.26 ± 0.24	1.19 ± 0.55	0.320
Left	1.20 ± 0.37	1.34 ± 0.36	1.08 ± 0.35	<0.001

Data are presented as mean ± standard deviation. Torque values are expressed as Nm/kg. H/Q ratios are dimensionless. Bilateral differences represent the percentage difference between the right and left limbs. The conventional H/Q ratio was calculated from concentric hamstring and quadriceps peak torque at the corresponding angular velocity. Group A, players without muscle strength imbalance; Group B, players with muscle strength imbalance.

### Primary outcome

During the 12-month follow-up, 40 of 134 players sustained at least one recorded hamstring injury, corresponding to a cumulative incidence of 29.9% (95% CI: 22.2–38.4). The cumulative incidence was 21.0% (13/62; 95% CI: 11.6–33.2) in Group A and 37.5% (27/72; 95% CI: 26.3–49.7) in Group B. Among the 40 injured players, injury was recorded on both the right and left sides in 22 players, on the left side only in 9 players, and on the right side only in 9 players.

### Factors associated with hamstring injury

In a single-predictor mixed-effects model accounting for club-level clustering, preseason isokinetic imbalance was associated with a higher odds of hamstring injury (OR: 2.12, 95% CI: 0.93–4.83; *p* = 0.073) (Table 3). After adjustment for previous hamstring injury and playing position, this association became statistically significant (OR: 2.56, 95% CI: 1.07–6.12; *p* = 0.034) (Table 4). In the multivariable model, a previous history of hamstring injury was also associated with higher odds of sustaining a subsequent hamstring injury (OR: 3.27, 95% CI: 1.20–8.93, *p* = 0.020). Playing position was not significantly associated with hamstring injury (OR: 0.90, 95% CI: 0.38–2.12, *p* = 0.808).

**Table 3:** Factors associated with hamstring injury after 12 months of prospective follow-up: single-predictor generalized linear mixed models accounting for club-level clustering.

Factors	No injury	Injury	OR [95% CI]	*p*
Group				
A (*n* = 62)	49 (79.0)	13 (21.0)	Ref.	
B (*n* = 72)	45 (62.5)	27 (37.5)	2.12 [0.93; 4.83]	0.073
Body mass index (kg/m^2^)	29.3 ± 3.1	30.1 ± 4.2	1.06 [0.94; 1.18]	0.325
Playing position				
Forwards (1–8) (*n* = 80)	56 (70.0)	24 (30.0)	Ref.	
Backs (9–15) (*n* = 54)	38 (70.4)	16 (29.6)	1.10 [0.50; 2.48]	0.808
Hamstring injury history				
No (*n* = 102)	75 (73.5)	27 (26.5)	Ref.	
Yes (*n* = 32)	19 (59.4)	13 (40.6)	2.65 [1.03; 6.81]	0.043
Extensibility of thigh muscles				
Right hamstring (degrees)	83.2 ± 10.4	82.1 ± 10.0	0.98 [0.94; 1.02]	0.359
Left hamstring (degrees)	81.9 ± 10.2	80.7 ± 10.7	0.99 [0.95; 1.03]	0.536
Right quadriceps (cm)	5 [0; 12]	11 [7; 15]	1.08 [0.98; 1.17]	0.102
Left quadriceps (cm)	6 [0; 12]	11 [6; 15]	1.07 [0.98; 1.16]	0.092

Data are presented as number of players (percentages in rows), mean ± standard deviation or median [25^th^; 75^th^ percentiles]. CI, confidence interval; Group A, players without muscle strength imbalance; Group B, players with muscle strength imbalance; OR, odds ratio; Ref, reference.

**Table 4:** Factors associated with hamstring injury after 12 months of prospective follow-up: multivariable generalized linear mixed model accounting for club-level clustering.

Factors	OR [95% CI]	*p*
Group		
A (*n* = 62)	Ref.	
B (*n* = 72)	2.56 [1.07; 6.12]	0.034
Hamstring injury history		
No (*n* = 102)	Ref.	
Yes (*n* = 32)	3.27 [1.20; 8.93]	0.020
Playing position		
Forwards (1–8) (*n* = 80)	Ref.	
Backs (9–15) (*n* = 54)	0.90 [0.38; 2.12]	0.808

CI, confidence interval; Group A, players without muscle strength imbalance; Group B, players with muscle strength imbalance; OR, odds ratio; Ref, reference.

The estimated between-club variance was 0.534, corresponding to an intraclass correlation coefficient of 0.140 (95% CI: 0.026–0.496). The likelihood-ratio test comparing the mixed-effects model with a standard logistic regression model was significant (*χ*^2^ = 9.03, *p* = 0.001), supporting the presence of club-level clustering. The proportion of players sustaining a hamstring injury was 28.0% (7/25) in Club 1, 53.3% (24/45) in Club 2, 13.8% (4/29) in Club 3, and 14.3% (5/35) in Club 4. Given the small number of participating clubs (*n* = 4), a sensitivity analysis was performed using standard logistic regression with club included as a fixed effect. The association between preseason isokinetic imbalance and subsequent hamstring injury remained in the same direction, although the estimate was less precise and did not reach conventional statistical significance (OR: 2.08, 95% CI: 0.90–4.81, *p* = 0.085).

The cumulative incidence of hamstring injury differed across the four subgroups defined by preseason isokinetic imbalance status and previous hamstring injury (*p* = 0.014). The cumulative incidence was 20.9% (9/43; 95% CI: 10.0–36.0) among Group A players without a history of hamstring injury, 21.1% (4/19; 95% CI: 6.1–45.6) among Group A players with a history of hamstring injury, 30.5% (18/59; 95% CI: 19.2–43.9) among Group B players without a history of hamstring injury, and 69.2% (9/13; 95% CI: 38.6–90.9) among Group B players with a history of hamstring injury.

The individual-level discriminative performance of the preseason isokinetic imbalance classification was subsequently assessed. The area under the receiver operating characteristic curve was 0.60 (95% CI: 0.51–0.69). Sensitivity and specificity were 67.5% (95% CI: 50.9–81.4) and 52.1% (95% CI: 41.6–62.5), respectively. The positive and negative predictive values were 37.5% (95% CI: 26.4–49.7) and 79.0% (95% CI: 66.8–88.3), respectively. The positive and negative likelihood ratios were 1.41 (95% CI: 1.04–1.91) and 0.62 (95% CI: 0.38–1.01), respectively.

## Discussion

The present prospective cohort study found that professional rugby union players classified with a preseason isokinetic muscle strength imbalance had higher odds of sustaining a hamstring injury during 12 months of follow-up after adjustment for previous hamstring injury and playing position. The estimated association increased from OR 2.12 (95% CI: 0.93–4.83) in the single-predictor mixed-effects model to OR 2.56 (95% CI: 1.07–6.12) after adjustment. This strengthening of the association may partly reflect the unequal distribution of previous hamstring injury between groups, as a history of hamstring injury was more frequent in Group A than in Group B (30.6% vs. 18.1%). However, this imbalance should not be interpreted as fully accounting for the difference between the single-predictor and adjusted estimates. Previous hamstring injury was also associated with subsequent hamstring injury after adjustment for preseason isokinetic imbalance and playing position (OR: 3.27, 95% CI: 1.20–8.93). The highest injury incidence was observed in players with both preseason isokinetic imbalance and previous hamstring injury (69.2%); however, this descriptive pattern should not be interpreted as evidence of an interaction. The association observed between preseason isokinetic imbalance and subsequent hamstring injury should be interpreted in the context of the heterogeneous literature on this topic. Croisier et al. reported a higher occurrence of hamstring injury among professional football players presenting untreated preseason strength imbalances and suggested that normalization of these abnormalities might reduce subsequent injury occurrence (19). Conversely, Bennell et al. found no predictive value of preseason isokinetic strength testing in Australian Rules football players (16). More recently, a systematic review and meta-analysis reported limited predictive validity of isokinetic strength assessments for detecting future hamstring injury (17), while a large prospective cohort study of professional football players concluded that a comprehensive strength-testing battery offered little clinical value for individual injury prediction (31). Taken together, these findings suggest that associations between strength characteristics and subsequent hamstring injury may vary according to the population studied, the measurement approach, and the sporting context. Therefore, the present results should not be interpreted as evidence that isokinetic testing alone provides valid individual-level prediction of future hamstring injury.

Previous hamstring injury remained associated with subsequent hamstring injury after adjustment for isokinetic imbalance and playing position, consistent with current evidence identifying previous injury as one of the most consistent risk factors for future hamstring injury 30. The cumulative incidence observed in our cohort was 29.9% (95% CI: 22.2–38.4). Direct comparison with published rugby injury studies should be undertaken cautiously because most studies report exposure-adjusted injury incidence rates or the proportion of all injuries represented by hamstring injuries, whereas the present study reports player-level cumulative incidence using a study-specific injury definition requiring more than two weeks of time loss (32). Nevertheless, 40 of 134 players sustained at least one qualifying hamstring injury during follow-up, indicating a substantial injury burden within this cohort.

Neither hamstring nor quadriceps extensibility was significantly associated with subsequent hamstring injury. Although greater heel-to-buttock distance was associated with a non-significant increase in injury odds, the study was likely underpowered to detect modest associations. Previous prospective studies evaluating flexibility and hamstring injury have reported inconsistent findings, potentially because of differences in sporting populations, assessment methods, injury definitions, and statistical approaches (3, 12, 33). Consequently, the absence of a statistically significant association in the present study should not be interpreted as evidence that flexibility is unrelated to hamstring injury.

An important consideration when interpreting these findings is the age of the dataset. Data were collected during the 2011–2012 sporting period, approximately 14 years before manuscript submission. Since then, professional rugby union has evolved substantially, with increased use of GPS-based workload monitoring (34), greater implementation of eccentric hamstring conditioning programmes (31), more standardized injury surveillance systems (32, 35), and contemporary approaches to hamstring injury diagnosis and management (36). Training load is also now recognized as an important factor associated with injury occurrence (37). Consequently, the present results should not be considered directly representative of the contemporary professional rugby environment. Rather, they provide historical prospective evidence supporting the need for replication in modern cohorts using contemporary injury definitions, exposure-adjusted analyses, and validated screening approaches.

### Clinical implications

The clinical implications of these findings should be interpreted cautiously. Although baseline isokinetic strength and muscle extensibility measures may be associated with subsequent hamstring injury, an observed association does not establish that these measures can accurately identify individual players at high risk of injury. Previous research has highlighted the limited predictive validity of isolated strength assessments, and the substantial overlap in test values between injured and non-injured athletes limits their discriminatory capacity at the individual level (25). In addition, hamstring injury is a multifactorial outcome influenced by previous injury, exposure to high-speed running, training and match load, fatigue, neuromuscular characteristics, and other individual and contextual factors (11).

Isokinetic assessment may nevertheless provide objective information on lower-limb strength characteristics that can contribute to the broader clinical assessment of professional rugby players. Any use of these measurements to guide individualized training should be considered hypothesis-generating rather than evidence of an effective injury-prevention strategy. Although corrective strength training was implemented for at least some players as part of routine club management, these interventions were not protocol-defined, standardized, or controlled, and their effect on subsequent hamstring injury occurrence could not be evaluated. Recent evidence in elite athletes has also suggested that isokinetic knee strength parameters may provide useful information for performance-oriented and injury-prevention-oriented training design, although the transferability of these findings to hamstring injury prevention in professional rugby remains uncertain (11).

In the present cohort, the limited individual-level discrimination of the isokinetic imbalance classification further supports this cautious interpretation. The sensitivity was 67.5%, specificity was 52.1%, positive predictive value was 37.5%, and negative predictive value was 79.0%. Future prospective studies should determine whether combining strength and extensibility measures with established injury-related factors and exposure measures improves individual-level discrimination, and controlled intervention studies are needed to determine whether targeting identified strength imbalances can reduce hamstring injury occurrence.

### Limitations

Several limitations should be considered when interpreting these findings. First, the age of the dataset limits the contemporary generalizability of the results. Data were collected during the 2011–2012 sporting period, approximately 14 years before submission of the present manuscript. The study was conducted according to a prospectively established protocol; however, no separate statistical analysis plan was prospectively registered, and some analyses and model specifications presented in the current manuscript were refined during peer-review revision. Professional rugby union has also evolved substantially since 2011–2012, including the widespread use of GPS-based exposure monitoring (34), greater implementation of eccentric hamstring conditioning programmes (31), changes in injury surveillance definitions, training and match loads and player rotation, tackle laws and collision profiles, increased use of synthetic playing surfaces, and greater integration of magnetic resonance imaging and structured clinical and functional criteria in hamstring injury management (36). These changes may have influenced both the prevalence of baseline strength abnormalities and the subsequent injury environment and therefore limit extrapolation of the present findings to contemporary professional rugby union (37).

In addition, players with a recent quadriceps or hamstring injury who had not returned to opposition training or match play for at least two weeks were excluded from preseason isokinetic testing. This exclusion may have removed players with residual strength deficits who were also at increased susceptibility to subsequent hamstring injury and may therefore have attenuated the observed association between preseason isokinetic imbalance and subsequent injury (11, 13). The exact number of players excluded for this reason was not available in the retained study records.

Measurement variability is another limitation. Isokinetic testing was performed using two different dynamometer models and by different physical therapists across the participating clubs. Although the physical therapists received standardized testing instructions and each dynamometer underwent its standard automatic calibration procedure before testing, no formal cross-calibration between the Cybex Norm® and Con-Trex MJ® devices was performed. Systematic inter-device differences in torque measurements therefore cannot be excluded (28, 29). No formal inter-rater reliability assessment was performed. Muscle extensibility assessments were also performed by physicians from the participating clubs, and no formal inter-rater reliability assessment was available for these measurements.

The classification of isokinetic imbalance also warrants cautious interpretation. The predefined cut-offs used in the present study were extrapolated from criteria previously described in professional football players (19) and have not been specifically validated in professional rugby union. Group B was defined by the presence of at least one predefined abnormality, including a side-to-side hamstring strength deficit >15%, an abnormal conventional H/Q ratio, or an abnormal mixed H/Q ratio. These abnormalities may reflect different underlying physiological characteristics, and the resulting composite group should therefore not be considered a homogeneous clinical phenotype. Although the frequency of each type of abnormality was available, individual-level information was insufficient to reliably reconstruct the overlap or combinations of abnormalities within Group B. No analysis of subsequent hamstring injury according to individual imbalance subtype or continuous-variable modelling of these measures was performed. Consequently, the present study cannot determine whether the observed association was driven predominantly by a particular type or magnitude of isokinetic abnormality.

A further limitation is the potential influence of uncontrolled co-interventions. Identified isokinetic abnormalities were communicated to the participating clubs, and corrective strength training was implemented for at least some affected players as part of routine clinical and performance management. However, the nature, timing, intensity, and adherence to these interventions were not systematically recorded. Their potential influence on subsequent hamstring injury occurrence therefore cannot be quantified. This uncontrolled co-intervention may have attenuated or otherwise modified the observed association between baseline isokinetic imbalance and subsequent injury, and the present study cannot establish whether correction of identified strength imbalances reduces hamstring injury occurrence.

Injury ascertainment was not blinded. Club medical staff had access to players' baseline isokinetic results and group allocation when monitoring subsequent injuries. Differential ascertainment cannot therefore be excluded, as awareness of a documented strength imbalance may have influenced clinical attention or attribution of subsequent hamstring events. In addition, the study-specific injury questionnaire was not formally validated and its reliability was not assessed.

The statistical analyses also have some limitations. The sample-size calculation was based solely on the expected precision of the primary descriptive endpoint; no formal sample-size or power calculation was performed for the secondary comparative analyses, which were considered exploratory. Furthermore, the limited number of injury events restricted the precision and statistical power of the comparative and multivariable analyses. The number of covariates was therefore restricted to limit model complexity and reduce the risk of overfitting, and playing position was collapsed from five categories into forwards and backs. Nevertheless, the number of events relative to the number of model parameters remained limited, and the adjusted estimates should be interpreted cautiously. In addition, only four clubs contributed data, resulting in a small number of clusters for estimating between-club variability in a random-effects framework. Estimates accounting for club-level clustering may therefore be imprecise and should also be interpreted with caution.

The discriminative performance of the baseline isokinetic classification was limited. Although preseason isokinetic imbalance was associated with higher odds of subsequent hamstring injury after adjustment for previous hamstring injury and playing position, the classification showed limited individual-level discrimination and should not be interpreted as a validated stand-alone screening or prediction tool. Further contemporary prospective studies are needed to determine whether rugby-specific isokinetic criteria, potentially combined with other established injury-related and exposure variables, can provide clinically useful individual-level discrimination.

Injury characterization was also limited by the study-specific definition of hamstring injury. A hamstring injury was defined as a clinically diagnosed injury resulting in more than two weeks of inability to participate in training or competition. Injuries associated with shorter periods of time loss were therefore not captured, and the study does not provide an estimate of the overall burden of hamstring injuries according to contemporary consensus definitions. Direct comparison with studies using lower time-loss thresholds is consequently limited. Although the study-specific injury questionnaire included items on injury date, recurrence status, injury circumstances and mechanism, phase of play, playing-surface conditions, and diagnostic investigations, the corresponding information was not sufficiently complete in the retained analytical dataset to allow reliable episode-level analyses. Consequently, first-time and recurrent hamstring injuries could not be reliably distinguished, contact-related injuries could not be differentiated from non-contact injuries, and the potential influence of playing-surface conditions or imaging findings could not be examined. Standardized injury severity grading was also not available.

Training and match exposure time was not collected. Consequently, injury occurrence could not be expressed as an exposure-adjusted incidence, nor could the analyses account for differences in training load, match participation, high-speed running exposure, or other workload-related factors between players. The temporal distribution of injuries throughout the season was also not analyzed. These limitations prevented a more precise assessment of whether injury occurrence varied according to exposure, competition density, cumulative workload, or the timing of the competitive season.

Age was not collected because of confidentiality requirements related to the participating professional clubs and could therefore neither be used to further characterize the cohort nor be considered as a potential confounding factor. Other potentially relevant individual or contextual characteristics were also not systematically available.

Finally, the ethics committee issued its favorable opinion on January 16, 2012, whereas recruitment had already begun for some participants. Consequently, some participants were included before the formal ethics committee opinion was obtained. This temporal discrepancy constitutes an important limitation of the study and should be considered when interpreting its ethical and regulatory framework.

## Conclusion

Approximately one in three professional rugby players in this cohort sustained a hamstring injury during the 12-month follow-up, using the study-specific definition of an injury resulting in more than two weeks of time loss. Baseline isokinetic muscle imbalance was associated with higher odds of subsequent hamstring injury after adjustment for previous hamstring injury and playing position. However, the single-predictor association was weaker, and the composite classification showed limited discrimination at the individual level. These findings should therefore be considered exploratory and require replication in contemporary professional rugby cohorts using standardized injury definitions, exposure-adjusted analyses, and validated, multidimensional screening approaches before their clinical relevance can be established.

## Data Availability

The raw data supporting the conclusions of this article will be made available by the authors, without undue reservation.
