# Supplementary material for: Preseason isokinetic strength imbalance and hamstring injury in male professional rugby players: a prospective cohort study
Source: Front Sports Act Living. 2026 Oct 28;8:1961372. doi: 10.3389/fspor.2026.1961372 (PMC13752200; doi:10.3389/fspor.2026.1961372)
Supplement: Supplementary file 1 [file Supplementaryfile1.docx]

**Injury history form**

**Remote from the knee (from the foot up to the lumbar spine)**

Anonymized Number: I__I - I__I__I__I__I

For each previous injury, please describe:

- The nature and location of the lesion (specify side):______________________________________
- Circumstances of the injury:________________________________________________________________
- Time since the injury occurred: ____________________________________________________________
- Duration of sports unavailability: __________________________________________________________

Injury History 1:

- ______________________________________________________________________________________________
- ______________________________________________________________________________________________
- ______________________________________________________________________________________________
- ______________________________________________________________________________________________

Injury History 2:

- ______________________________________________________________________________________________
- ______________________________________________________________________________________________
- ______________________________________________________________________________________________
- ______________________________________________________________________________________________

Injury History 3:

- ______________________________________________________________________________________________
- ______________________________________________________________________________________________
- ______________________________________________________________________________________________
- ______________________________________________________________________________________________

Injury History 4:

- ______________________________________________________________________________________________
- ______________________________________________________________________________________________
- ______________________________________________________________________________________________
- ______________________________________________________________________________________________

**Injury history form (knee region)**

Anonymized Number: |__| - |__|__|__|__|

**Injury Record 1**:

□ Muscle injury

Specify the muscle:	□ Hamstrings	□ Quadriceps

Side:	□ Right	□ Left

Type of injury:	□ Intrinsic	□ Extrinsic

Date of injury: _________________________________________

Recurrence?	□ No	□ Yes – Date of most recent recurrence:

Time off sport:	□ 2 weeks < “Duration” < 4 weeks

□ 4 weeks < “Duration” < 6 weeks

□ “Duration” ≥ 6 weeks

□ Tendon injury – specify the tendon:

□ Tendinopathy	Tendon rupture:	□ Partial	□ Complete

Ligament injury – specify the ligament:

□ ACL	□ PCL	□ MCL	□ LCL

□ Sprain	Rupture:	□ Partial	□ Complete

Other injuries – specify:_________________________________________________________________________

**What treatments were used**?

□ Physical therapy	□ Surgery

**Was isokinetic testing used in rehabilitation?**	□ Yes	□ No

**How does the player rate their current functional recovery?**

□ Very good	□ Good	□ Average	□ Poor

**If recovery is average or unsatisfactory, what symptoms persist?**__________________________________________________________________________________________________________________________________________________________________________________________________________________________________________________________________________________________________________________

**Injury Record 2:**

□ Muscle injury

Specify the muscle:	□ Hamstrings	□ Quadriceps

Side:	□ Right	□ Left

Type of injury:	□ Intrinsic	□ Extrinsic

Date of injury: _________________________________________

Recurrence?	□ No	□ Yes – Date of most recent recurrence:

Time off sport:	□ 2 weeks < “Duration” < 4 weeks

□ 4 weeks < “Duration” < 6 weeks

□ “Duration” ≥ 6 weeks

□ Tendon injury – specify the tendon:

□ Tendinopathy	Tendon rupture:	□ Partial	□ Complete

Ligament injury – specify the ligament:

□ ACL	□ PCL	□ MCL	□ LCL

□ Sprain	Rupture:	□ Partial	□ Complete

Other injuries – specify:_________________________________________________________________________

**What treatments were used**?

□ Physical therapy	□ Surgery

**Was isokinetic testing used in rehabilitation?**	□ Yes	□ No

**How does the player rate their current functional recovery?**

□ Very good	□ Good	□ Average	□ Poor

**If recovery is average or unsatisfactory, what symptoms persist?**__________________________________________________________________________________________________________________________________________________________________________________________________________________________________________________________________________________________________________________

**Injury Record 3:**

□ Muscle injury

Specify the muscle:	□ Hamstrings	□ Quadriceps

Side:	□ Right	□ Left

Type of injury:	□ Intrinsic	□ Extrinsic

Date of injury: _________________________________________

Recurrence?	□ No	□ Yes – Date of most recent recurrence:

Time off sport:	□ 2 weeks < “Duration” < 4 weeks

□ 4 weeks < “Duration” < 6 weeks

□ “Duration” ≥ 6 weeks

□ Tendon injury – specify the tendon:

□ Tendinopathy	Tendon rupture:	□ Partial	□ Complete

Ligament injury – specify the ligament:

□ ACL	□ PCL	□ MCL	□ LCL

□ Sprain	Rupture:	□ Partial	□ Complete

Other injuries – specify:_________________________________________________________________________

**What treatments were used**?

□ Physical therapy	□ Surgery

**Was isokinetic testing used in rehabilitation?**	□ Yes	□ No

**How does the player rate their current functional recovery?**

□ Very good	□ Good	□ Average	□ Poor

**If recovery is average or unsatisfactory, what symptoms persist?**__________________________________________________________________________________________________________________________________________________________________________________________________________________________________________________________________________________________________________________

**DATA COLLECTION**

**(History, FLEXIBILITY, ISOKINETIC TESTS)**

Anonymized number: I__I - I__I__I__I__I

Weight: _________ Kg

Height: __________ cm

Position (for front row, specify Right, Left or both): ______________________________________________

1. Previous injuries:

- Near the knee (list only the lesion; detailed description on the nearby knee injury sheet).
  - _____________________________________________________________________________________
  - _____________________________________________________________________________________
  - _____________________________________________________________________________________
  - _____________________________________________________________________________________
- Remote from the knee (list only the lesion; detailed description on the remote knee injury sheet).
  - _____________________________________________________________________________________
  - _____________________________________________________________________________________
  - _____________________________________________________________________________________
  - _____________________________________________________________________________________

2. Flexibility

|  | Left side | Right side |
| --- | --- | --- |
| Hip flexion, knee extended (in degrees) (Hamstrings) |  |  |
| Heel-to-buttock distance (in cm) (Quadriceps) |  |  |
| Knee flexion angle in prone position (Quadriceps) |  |  |

3. Isokinetic tests

Please specify the type of isokinetic device used:_________________________________________________

**Prospective injury collection near the knee**

Anonymized number: I__I - I__I__I__I__I

**Nature of the lesion (location and severity)?**

🞎 Muscle injury – specify the muscle:

🞎 Hamstrings	🞎 Quadriceps

Side:	🞎 Right	🞎 Left

Type of injury: 🞎Intrinsic	🞎 Extrinsic

🞎 Tendon injury – specify the tendon:

🞎 Tendinopathy	Tendon rupture: 🞎Partial	🞎 Complete

🞎 Ligament injury – specify the ligament:

🞎ACL	🞎PCL	🞎 MCL	🞎LCL

🞎Sprain	Rupture: 🞎Partial

🞎Complete

🞎Other injuries – specify:____________________________________________________________________________

**What treatments were used**?	🞎 Physical therapy	🞎 Surgery

**Use of isokinetics in rehabilitation**: 🞎 Yes	🞎 No

**How does the player rate their recovery in current activity**?

🞎 Very good	🞎 Good	🞎 Average	🞎 Poor

**Circumstances of the injury:**

🞎 With contact	🞎Without contact

**Game phase**:

🞎Running	🞎 Kicking	🞎 Tackling	🞎 Ruck/maul

🞎 Other: specify

**Time of injury**:

🞎Warm-up	🞎1st quarter	🞎 2nd quarter	🞎 3rd quarter	🞎4th quarter

**Field condition**: 🞎 Hard	🞎Normal	🞎Muddy	🞎Soaked

**What was the player's position at the time of injury?** (indicate with a number from 1 to 15) _________________________________________________________________________________________________

**Time interval between the injury and the isokinetic evaluation in this stud**y? (in weeks) _____________________________________________________________________________________________

**What tests were performed for diagnosis?** (check all that apply)

🞎Clinical exam	🞎 CT scan

🞎X-rays	🞎Arthro-CT

🞎 Ultrasound	🞎 MRI

🞎Other, specify: ___________________________________________________________________

**What criteria were used to resume sport?** (check all that apply)

🞎Clinical exam

🞎Imaging, specify: _____________________________________________________________

🞎 Field tests

🞎Isokinetic evaluation
