# Supplementary material for: Preseason isokinetic strength imbalance and hamstring injury in male professional rugby players: a prospective cohort study
Source: Front Sports Act Living. 2026 Oct 28;8:1961372. doi: 10.3389/fspor.2026.1961372 (PMC13752200; doi:10.3389/fspor.2026.1961372)
Supplement: Supplementary file 2 [file Table1.docx]

**Supplementary Table S1.** Detailed isokinetic testing protocol

| **Parameters** | **Protocol** |
| --- | --- |
| Isokinetic dynamometer | Con-Trex® or Cybex Norm®, according to the participating club |
| Device calibration | Standard automatic calibration was performed before each assessment according to the manufacturer's instructions |
| Testing position | Seated position, seat raised and backrest inclined at 85° |
| Pre-test warm-up | 10 min of cycling on an ergometric bicycle at approximately 1 W/kg |
| Range of motion | 100° of knee flexion from maximal active extension, without recurvatum |
| Contraction mode | Concentric and eccentric |
| Concentric muscle groups | Quadriceps and hamstrings |
| Concentric angular velocities | 60°/s and 240°/s |
| Repetitions at 60°/s | 3 familiarization repetitions followed by 3 maximal repetitions |
| Repetitions at 240°/s | 3 familiarization repetitions followed by 5 maximal repetitions |
| Eccentric muscle group | Hamstrings only |
| Eccentric angular velocity | 30°/s and 120°/s |
| Eccentric testing at 30°/s | 3 familiarization repetitions, or additional repetitions when necessary, followed by 3 maximal repetitions |
| Eccentric testing at 120°/s | 3 familiarization repetitions followed by 4 maximal repetitions |
| Eccentric quadriceps testing | Not performed |
| Value retained for analysis | Maximum peak torque (MPT) |
| Torque normalization | Normalized to body mass |
| Bilateral assessment | Difference between right and left limbs |
| H/Q ratio | Conventional H/Q ratio calculated from concentric peak torque |
| Rest interval between testing sets | 1 min |
| General familiarization | 6–7 submaximal repetitions followed by 3–4 progressively maximal repetitions at 120°/s before the maximal testing protocol; these repetitions were not recorded for analysis |
| Visual feedback | Not provided during testing |
| Standardization | All physical therapists performing the assessment received standardized testing instructions |

**Abbreviations:** H: hamstrings; Q: quadriceps; H/Q: hamstring-to-quadriceps ratio; MPT: maximum peak torque.

Note: Eccentric testing was performed for the hamstrings only. Therefore, an eccentric conventional H/Q ratio could not be calculated. The eccentric hamstring assessment at 120°/s was performed as part of the original testing protocol but was not used to define the eccentric side-to-side hamstring strength criterion reported in the present study.
