# Supplementary material for: Preseason isokinetic strength imbalance and hamstring injury in male professional rugby players: a prospective cohort study
Source: Front Sports Act Living. 2026 Oct 28;8:1961372. doi: 10.3389/fspor.2026.1961372 (PMC13752200; doi:10.3389/fspor.2026.1961372)
Supplement: Supplementary file 3 [file Table2.docx]

**Supplementary Table S2**. Absolute peak torque values and H/Q ratios according to preseason isokinetic imbalance status.

|  | **Total**  (n=134) | **Group A**  (n=62) | **Group B**  (n=72) | p |
| --- | --- | --- | --- | --- |
| Peak concentric hamstring torque 60°/s |  |  |  |  |
| Right | 164 ± 27 | 173 ± 23 | 155 ± 28 | <0.001 |
| Left | 164 ± 29 | 174 ± 25 | 156 ± 30 | <0.001 |
| Peak concentric quadriceps torque 60°/s |  |  |  |  |
| Right | 264 ± 49 | 275 ± 45 | 255 ± 51 | 0.020 |
| Left | 261 ± 50 | 269 ± 51 | 254 ± 48 | 0.090 |
| Peak concentric hamstring torque 240°/s |  |  |  |  |
| Right | 127 ± 24 | 130 ± 24 | 125 ± 24 | 0.260 |
| Left | 128 ± 30 | 131 ± 30 | 126 ± 30 | 0.290 |
| Peak concentric quadriceps torque 240°/s |  |  |  |  |
| Right | 180 ± 33 | 180 ± 28 | 179 ± 38 | 0.920 |
| Left | 178 ± 31 | 176 ± 33 | 180 ± 30 | 0.450 |
| Peak eccentric hamstring torque 30°/s |  |  |  |  |
| Right | 210 ± 39 | 223 ± 35 | 198 ± 38 | <0.001 |
| Left | 207 ± 48 | 227 ± 36 | 190 ± 51 | <0.001 |
| Conventional H/Q ratio 60°/s |  |  |  |  |
| Right | 0.63 ± 0.15 | 0.64 ± 0.10 | 0.63 ± 0.19 | 0.710 |
| Left | 0.64 ± 0.14 | 0.67 ± 0.15 | 0.62 ± 0.12 | 0.070 |
| Mixed H/Q ratio |  |  |  |  |
| Right | 1.22 ± 0.43 | 1.26 ± 0.24 | 1.19 ± 0.55 | 0.320 |
| Left | 1.20 ± 0.37 | 1.34 ± 0.36 | 1.08 ± 0.35 | <0.001 |
| *Data are presented as mean ± standard deviation. Group A: players without muscle strength imbalance; Group B: players with muscle strength imbalance. Torque values are expressed as Nm. H/Q ratios are dimensionless.* | | | | |
